# Supplementary material for: Reciprocal Changes in miRNA Expression with Pigmentation and Decreased Proliferation Induced in Mouse B16F1 Melanoma Cells by l-Tyrosine and 5-Bromo-2′-Deoxyuridine
Source: Int J Mol Sci. 2021 Feb 5;22(4):1591. doi: 10.3390/ijms22041591 (PMC7914888; doi:10.3390/ijms22041591)
Supplement: Supplementary file 1 [file ijms-22-01591-s001.zip › Supplementary material.docx]

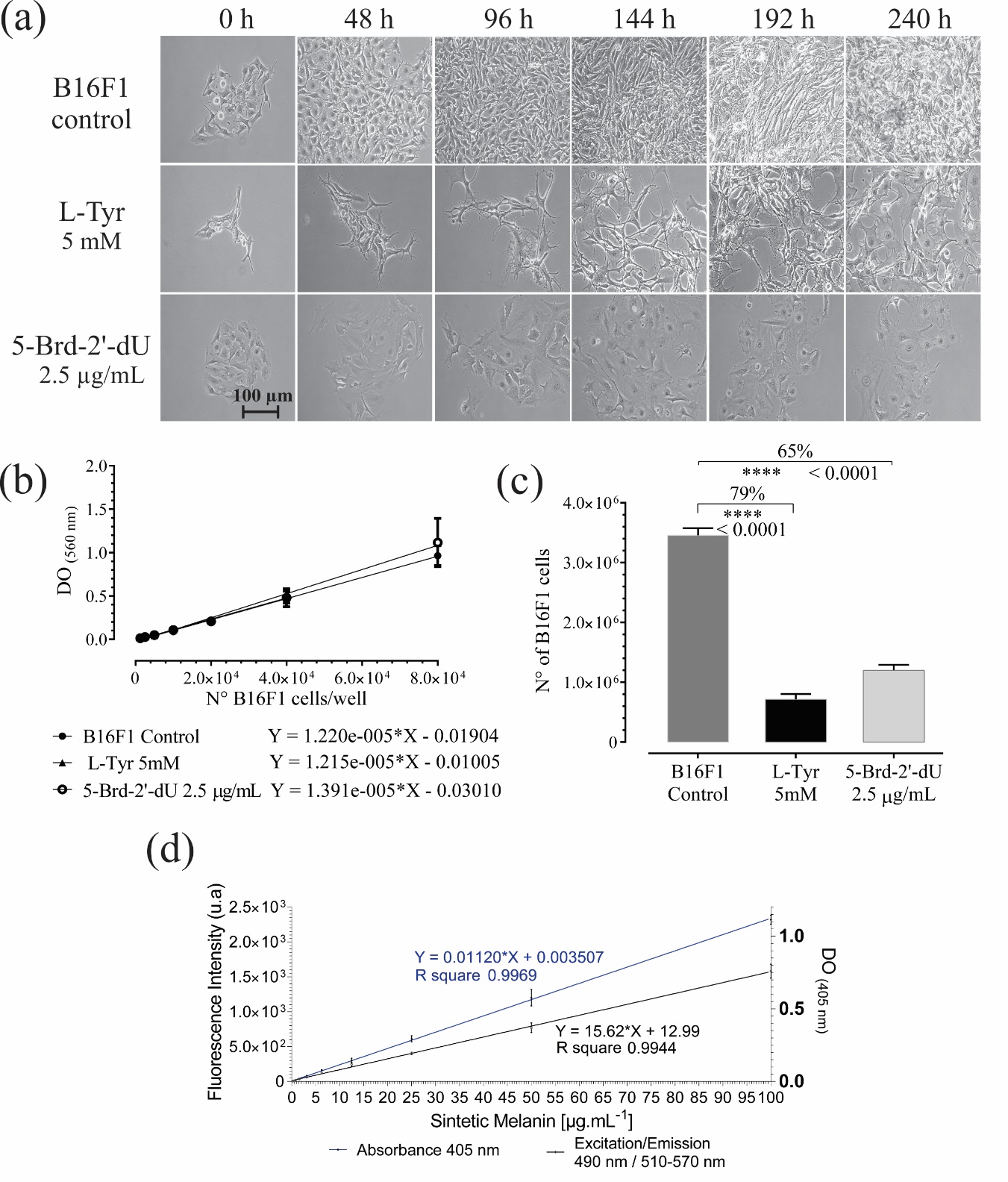


**Figure S1.** (**a**) Representative photographs of B16F1 cells between 0 h and 240 h after exposure to 5 mM L-Tyr or 2.5 µg. mL-1 BrdU. (**b**) Calibration curve for B16F1 cells and their absorbance measured by MTT reductase activity. (**c**) Number of B16F1 cells determined by MTT reductase activity after exposure to L-Tyr or BrdU. (**d**) Calibration curve for synthetic melanin and its quantification by absorbance and fluorescence. We considered statistically significant (*) using a two-tailed t-Student test, and the difference for a *p-value* <0.05, ns: not significant.


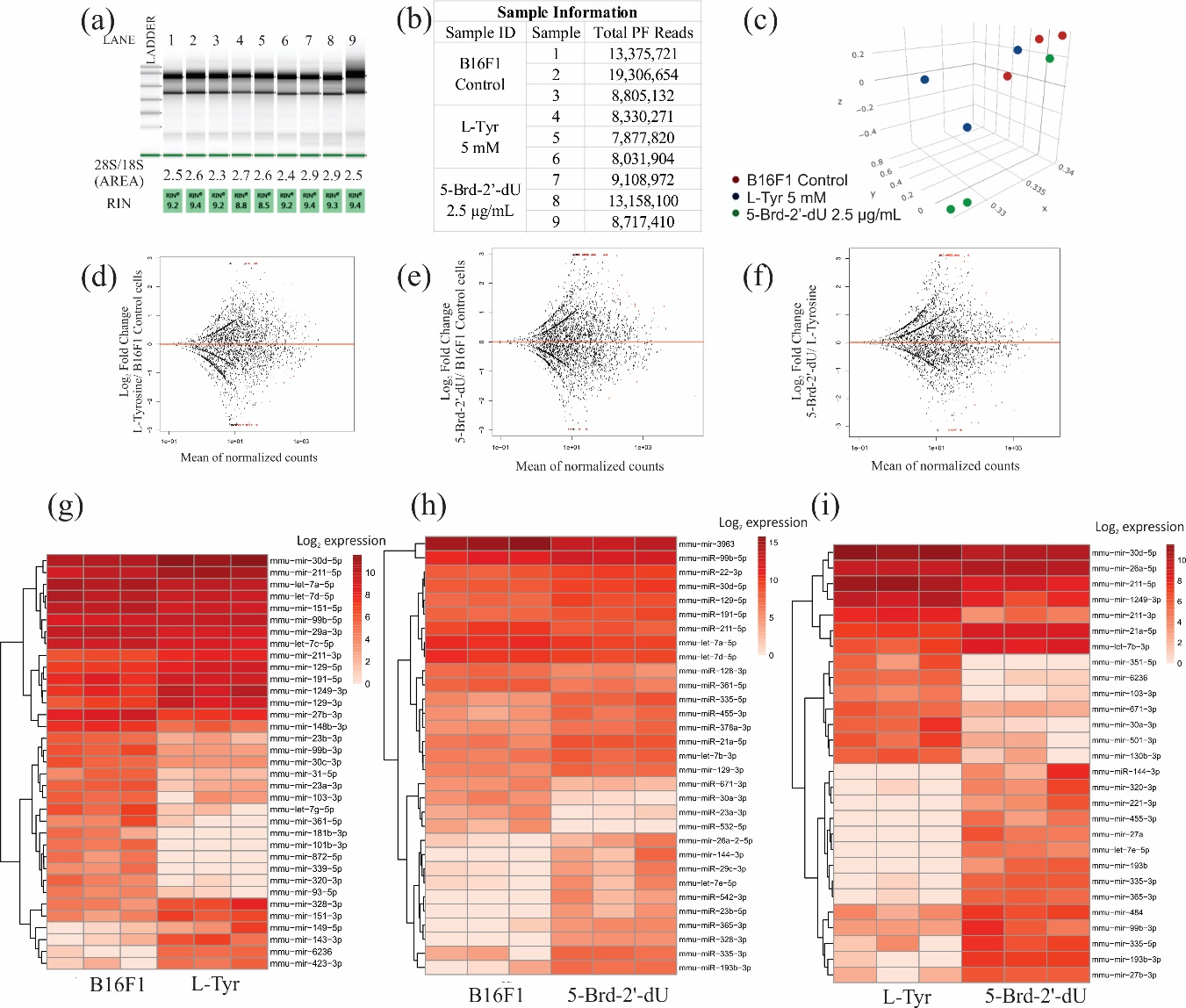


**Figure S2.** (**a**) Capillary electrophoresis and RNA integrity number (RIN) for RNA samples extracted from unexposed B16F1 cells (1, 2, and 3), exposed to L-Tyr (4, 5, and 6) or BrdU (7, 8, and 9). (**b**) The total number of reads obtained by last generation sequencing (small RNAseq). (**c**) Principal Component Analysis (PCA) in 3D for the total reads obtained by small RNAseq. (**d-f**) A plot of the logarithm of the fold change concerning the mean of normalized counts for the miRNAs obtained by sequencing in melanoma cells exposed to L-Tyr (**d**), BrdU (**e**), and BrdU concerning L-Tyr (**f**). (**g-i**) A heat map with the relative expression levels of miRNAs differentially expressed in B16F1 cells exposed to L-Tyr (**g**) and BrdU (**h**) concerning non-exposed cells and in (**i**) the expression of the BrdU miRNAs concerning L -Tyr. Increased intensity of the red color indicates overexpression.

**Table S1.** Functional Enrichment using KEGG on the microRNAs-target regulation network (L-Tyrosine up and down-regulated concerning B16F1 control cells)

**Table S2.** Functional Enrichment using KEGG on the microRNAs-target regulation network (BrdU up and down-regulated concerning B16F1 control cells)

**Table S3.** Functional Enrichment using KEGG on the microRNAs-target regulation network (BrdU up and down-regulated concerning L-Tyr)

**Table S4.** List of primer sets for genes and miRNAs used in RT-qPCR and RT-qPCR- stem loop
