# Supplementary material for: Reciprocal Changes in miRNA Expression with Pigmentation and Decreased Proliferation Induced in Mouse B16F1 Melanoma Cells by l-Tyrosine and 5-Bromo-2′-Deoxyuridine
Source: Int J Mol Sci. 2021 Feb 5;22(4):1591. doi: 10.3390/ijms22041591 (PMC7914888; doi:10.3390/ijms22041591)
Supplement: Supplementary file 1 [file ijms-22-01591-s001.zip › Supplementary Table S4. Primers list/Supplementary Table S4. Primers list.pdf]

Gene - miRNA name	Direction	Sequence (5´ to 3´)	Annealing temperature (ºC)
Microphthalmia-associated	Forward	agg acc ttg aaa acc gac ag	59
	Reverse	gtg gat ggg ata agg gaa ag	
Tyrosinase	Forward	agc ctg tgc ctc ctc taa	56
	Reverse	agg aac ctc tgc ctg aaa	
Tyrosinase-related protein 1	Forward	cga tac cct ggg aac act	56
	Reverse	tac acg gac ctc caa gca	
Dopachrome Tautomerase	Forward	cca acg ctg att agt cgg a	57
	Reverse	gaa gaa ggg agg gct gtc a	
Cyclin-dependent kinase inhibitor 1A -	Forward	aac atc tca ggg ccg aaa ac	68
	Reverse	taa gac aca cag agt gag gg	
Cyclin D1	Forward	tgc atg ttc gtg gcc tct aa	65
	Reverse	ctt gcg gat ggt ctg ctt gt	
Cyclin-dependent kinase 4	Forward	aca agt aat ggg acc gtc aa	62
	Reverse	ggt cgg ctt cag agt ttc ca	
Cyclin-dependent kinase 2	Forward	aga ggg cac gta cgg agt	64
	Reverse	tgg agc agc tgg aac aga ta	
Glyceraldehyde-3-Phosphate	Forward	acc aca gtc cat gcc atc act	------------
	Reverse	acc agg aaa tga gct tga caa agt	
mmu-miR-27b-3p	Stem-loop	gtc gta tcc agt gca ggg tcc gag gta ttc gca ctg gat acg acg cag aa	60
	Forward	cgg ctt cac agt ggc taa g	
mmu-miR-148b-3p	Stem-loop	gtc gta tcc agt gca ggg tcc gag gta ttc gca ctg gat ac gac aca aag	
	Forward	cgg ctt cac agt ggc taa g	
mmu-miR-211-5p	Stem-loop	gtc gta tcc agt gca ggg tcc gag gta ttc gca ctg gat acg aca ggc aa	
	Forward	cgg ctt ccc ttt gtc atc c	
mmu-miR-129-5p	Stem-loop	gtc gta tcc agt gca ggg tcc gag gta ttc gca ctg gat acg acg caa gc	
	Forward	cgg cct ttt gcg gtc tgg	
mmu-miR-30d-5p	Stem-loop	gtc gta tcc agt gca ggg tcc gag gta ttc gca ctg gat acg acc ttc ca	
	Forward	cgg ctg taa aca tcc ccg a	
mmu-miR-470-5p	Stem-loop	gtc gta tcc agt gca ggg tcc gag gta ttc gca ctg gat acg aca ctc ac	
	Forward	gcc gtt ctt gga ctg gca ctg	
mmu-miR-470-3p	Stem-loop	gtc gta tcc agt gca ggg tcc gag gta ttc gca ctg gat acg act ctt ct	
	Forward	gcc gga acc agt acc ttt ct	
Universal Reverse	------------	gtg cag ggt ccg agg t	
Small nucleolar RNA U6	Stem-loop	gtc gta tcc agt gca ggg tcc gag gta ttc gca ctg gat acg aca aat atg	
	Forward	tgg aac gat aca gag aag att tag ca	
	Reverse	aac gct tca cga att tgc gt	
