# Supplementary figures and images for: Reciprocal Changes in miRNA Expression with Pigmentation and Decreased Proliferation Induced in Mouse B16F1 Melanoma Cells by l-Tyrosine and 5-Bromo-2′-Deoxyuridine
Source: Int J Mol Sci. 2021 Feb 5;22(4):1591. doi: 10.3390/ijms22041591 (PMC7914888; doi:10.3390/ijms22041591)

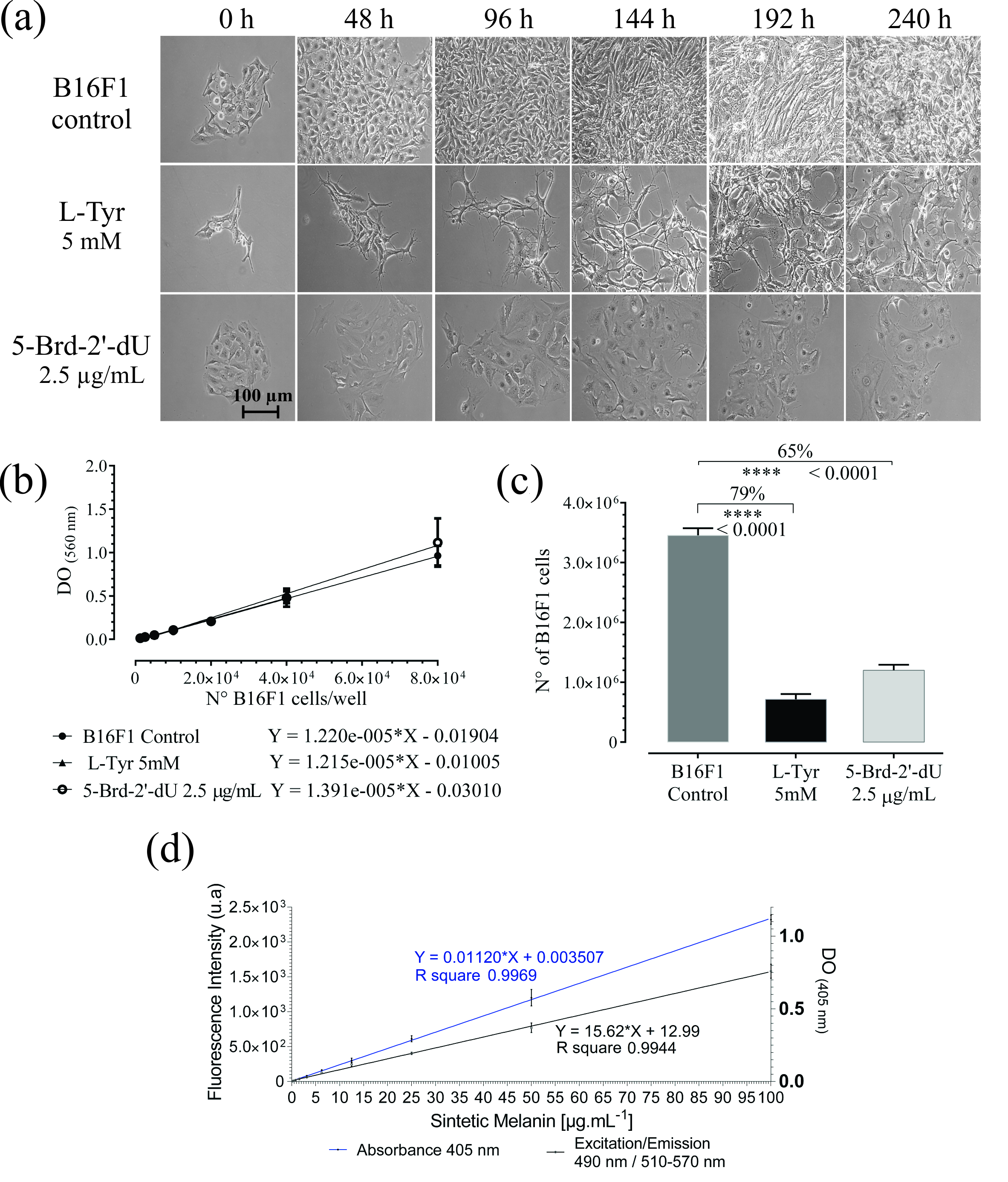

Supplement: Supplementary file 1 [file ijms-22-01591-s001.zip › FIGURE 1S.jpg]

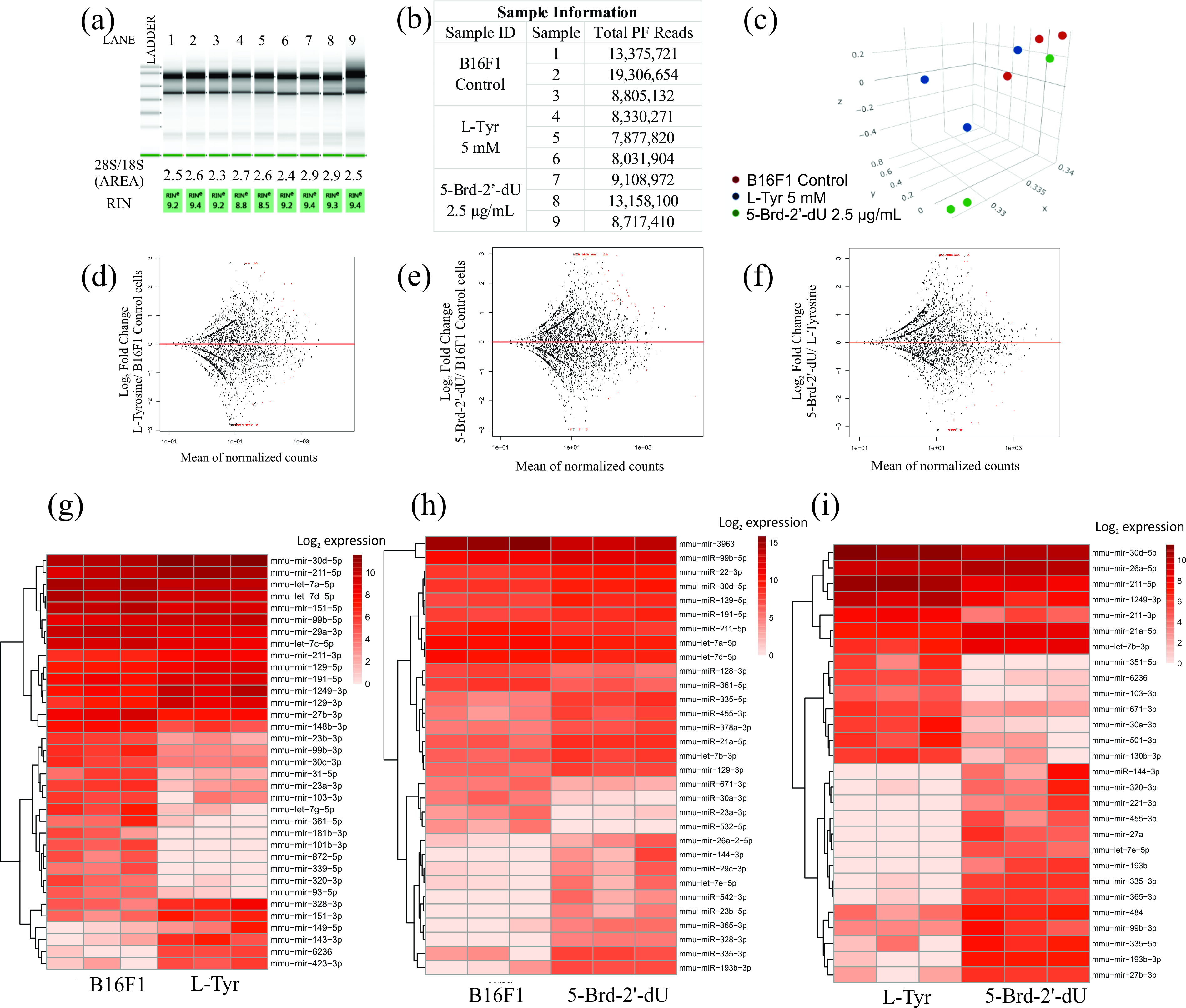

Supplement: Supplementary file 1 [file ijms-22-01591-s001.zip › FIGURE 2S.jpg]

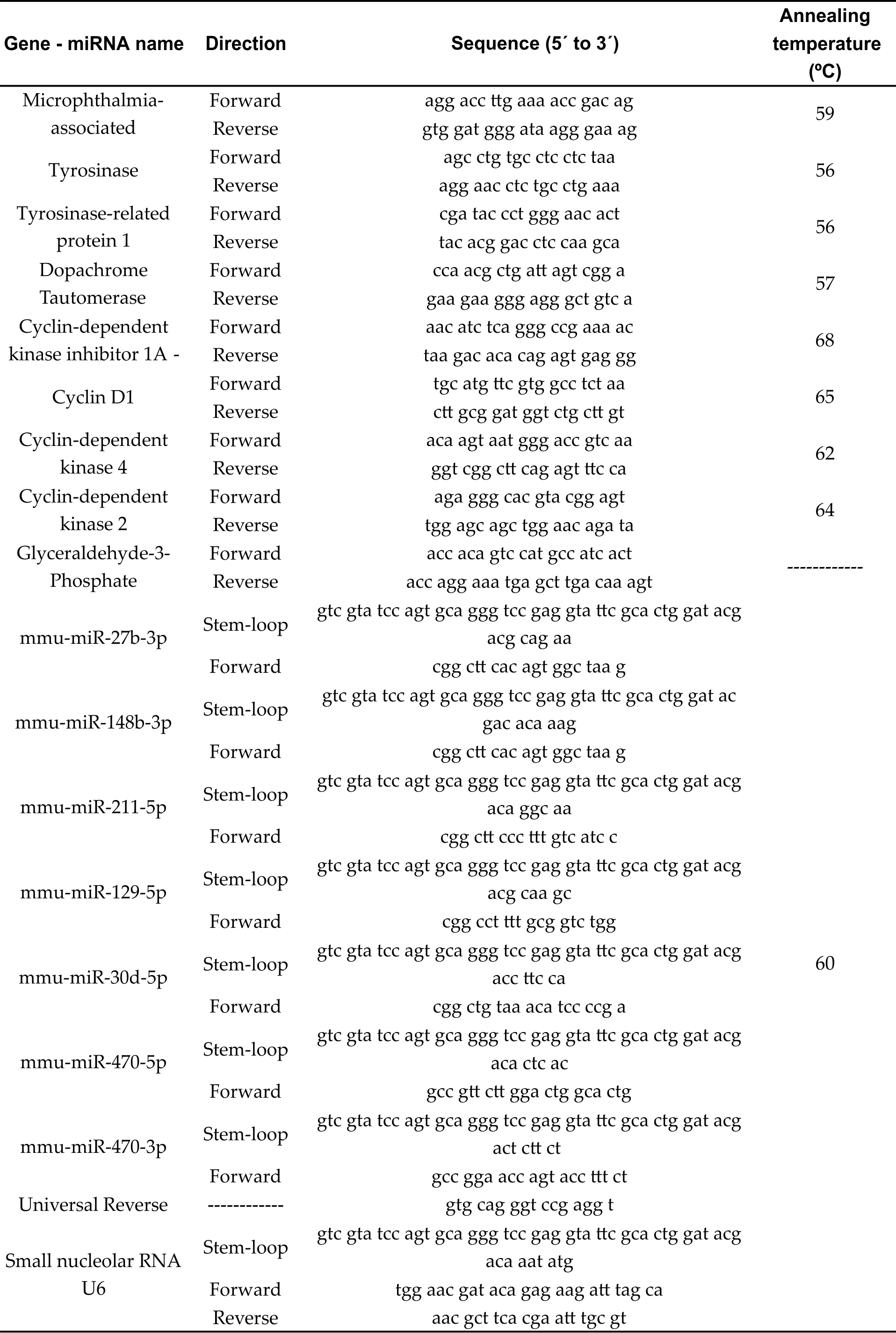

Supplement: Supplementary file 1 [file ijms-22-01591-s001.zip › Supplementary Table S4. Primers list/Supplementary Table S4. Primers list.jpg]
